# Algorithm-based detection of acute kidney injury according to full KDIGO criteria including urine output following cardiac surgery: a descriptive analysis

**DOI:** 10.1186/s13040-023-00323-3

**Published:** 2023-03-16

**Authors:** Nico Schmid, Mihnea Ghinescu, Moritz Schanz, Micha Christ, Severin Schricker, Markus Ketteler, Mark Dominik Alscher, Ulrich Franke, Nora Goebel

**Affiliations:** 1 Department of Medical Informatics, Robert Bosch Society for Medical Research, Stuttgart, Germany; 2 Department of Cardiovascular Surgery, Robert Bosch Hospital, Stuttgart, Germany; 3 Division of General Internal Medicine and Nephrology, Department of Internal Medicine, Robert Bosch Hospital, Stuttgart, Germany; 4 Executive Chief Physician of Robert Bosch Hospital and director of Robert Bosch Society for Medical Research, Stuttgart, Germany

**Keywords:** Acute kidney injury, Cardiac surgery, Intensive care, Automated detection, Big data analysis, Algorithm-based detection

## Abstract

**Background:**

Automated data analysis and processing has the potential to assist, improve and guide decision making in medical practice. However, by now it has not yet been fully integrated in a clinical setting. Herein we present the first results of applying algorithm-based detection to the diagnosis of postoperative acute kidney injury (AKI) comprising patient data from a cardiac surgical intensive care unit (ICU).

**Methods:**

First, we generated a well-defined study population of cardiac surgical ICU patients by implementing an application programming interface (API) to extract, clean and select relevant data from the archived digital patient management system. Health records of *N* = 21,045 adult patients admitted to the ICU following cardiac surgery between 2012 and 2022 were analyzed. Secondly, we developed a software functionality to detect the incidence of AKI according to Kidney Disease: Improving Global Outcomes (KDIGO) criteria, including urine output. Incidence, severity, and temporal evolution of AKI were assessed.

**Results:**

With the use of our automated data analyzing model the overall incidence of postoperative AKI was 65.4% (*N* = 13,755). Divided by stages, AKI 2 was the most frequent maximum disease stage with 30.5% of patients (stage 1 in 17.6%, stage 3 in 17.2%). We observed considerable temporal divergence between first detections and maximum AKI stages: 51% of patients developed AKI stage 2 or 3 after a previously identified lower stage. Length of ICU stay was significantly prolonged in AKI patients (8.8 vs. 6.6 days, *p* <  0.001) and increased for higher AKI stages up to 10.1 days on average. In terms of AKI criteria, urine output proved to be most relevant, contributing to detection in 87.3% (*N* = 12,004) of cases.

**Conclusion:**

The incidence of postoperative AKI following cardiac surgery is strikingly high with 65.4% when using full KDIGO-criteria including urine output. Automated data analysis demonstrated reliable early detection of AKI with progressive deterioration of renal function in the majority of patients, therefore allowing for potential earlier therapeutic intervention for preventing or lessening disease progression, reducing the length of ICU stay, and ultimately improving overall patient outcomes.

**Graphical Abstract:**

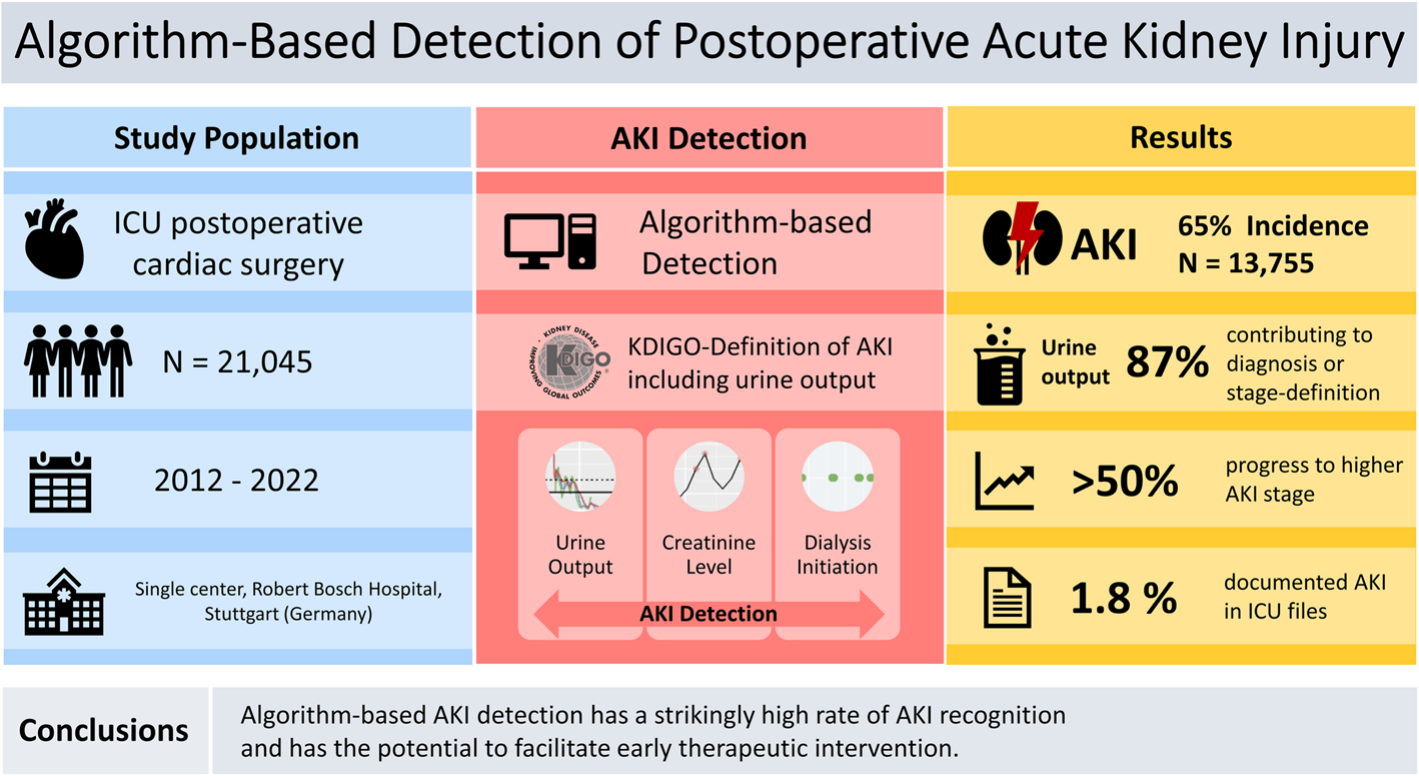

**Supplementary Information:**

The online version contains supplementary material available at 10.1186/s13040-023-00323-3.

## Background

The application of tools from modern medical informatics to existing data sets from routine care is an emerging field in medicine. Promises in this area range from the improvement in diagnosing conditions with subtle clinical representation to an accurately description of patient populations. Basis for such improvements is the automated extraction and accurate analysis of this data in a privacy-compliant manner [1].

Furthermore, automated data extraction from an existing database can serve as a solid basis for training machine learning algorithms. These have proven to be an exceedingly useful tool in the clinical setting over the past years with regards to early diagnosis, recognizing developing complications and ultimately improving patient outcomes especially in an intensive care setting [2].

Diagnosis itself is partially subjective and often based on subjective reasoning, therefore directly dependent on the physician and individual experience. This integrated process will most likely never become fully automated, at least not in the near future. On the other hand automated data processing can assist the clinician in arranging, and highlighting the relevant information in a timely manner. Current efforts are geared toward reducing the physicians’ workload and minimizing human error [3].

The current project relates to the implementation of a software to automatically detect AKI according to full KDIGO-criteria including urine output in a postoperative ICU-setting following cardiac surgery.

It’s known that AKI is associated with a high mortality rate up to 60% on ICU and up to 1 year after discharge, making early detection and prevention crucial [4–7]. In the last 50 years, the mortality of ICU patients on kidney replacing therapy (KRT) has unfortunately not significantly improved and remains very high [8]. Although the incidence of AKI is high and associated with worse outcomes, it is often underestimated and frequently under-reported [9]. In addition, reported AKI rates show wide variations as the diagnosis is often based on serum creatinine values alone as is often seen in studies of automated AKI detection [10]. However, not considering urine output in the diagnosis of AKI can significantly underestimate incidence and mortality [11].

Previous studies state that automated AKI detection and prediction can outperform human predictive performances [3].

In this study, we performed a retrospective descriptive analysis of a monocentric patient cohort at a cardiac center in southern Germany. The series includes *N* = 21,045 intensive care patients undergoing cardiac surgery and was designed to include data on individual patient condition, comorbidities, and aggravating factors in addition to static and dynamic parameters and medication. The main objective of this work was, besides a feasibility analysis regarding the application of algorithm-based AKI detection on already existing data, caption of the true AKI incidence according to full KDIGO criteria including urine output of cardiac surgery ICU patients and a description of the generated cohort.

This holds great potential to provide insights into hospital processes and AKI disease progression. Furthermore, reliable detection is a fundamental cornerstone for implementing an artificial intelligence (AI) based prevention program that could ultimately help in early prediction of AKI onset using real-time data.

## Methods

### Endpoint definition of acute kidney injury

For detecting AKI at any stage, we followed the international definition of AKI according to the KDIGO criteria and implemented a corresponding software functionality (see Table 1). AKI is assumed when at least one of the defined criteria, that is, increased serum creatinine level, decreased urine output or initiation of KRT, are met in an independent manner. Whenever more than one criterion simultaneously indicates a disease, only the higher AKI stage is considered in further analysis.

**Table 1:** KDIGO definition of AKI

Stage	Creatinine	Urine output	Dialysis
AKI 1	baseline multiplier ≥1.5 **AND** ≤ 1.9 **OR** ≥ 0.3 mg/dl increase	< 0.5 ml/kg/h for 6-12 h	
AKI 2	baseline multiplier ≥2.0 **AND** ≤ 2.9	< 0.5 ml/kg/h for ≥12 h	
AKI 3	baseline multiplier ≥3.0 **OR** ≥ 4.0 mg/dl **AND** ≥ 0.5 mg/dl increase	< 0.3 ml/kg/h for 24 h–48 h	initiation of dialysis

Definition of AKI according to KDIGO definition including urine output

### Data collection and processing

**Fig. 1:**
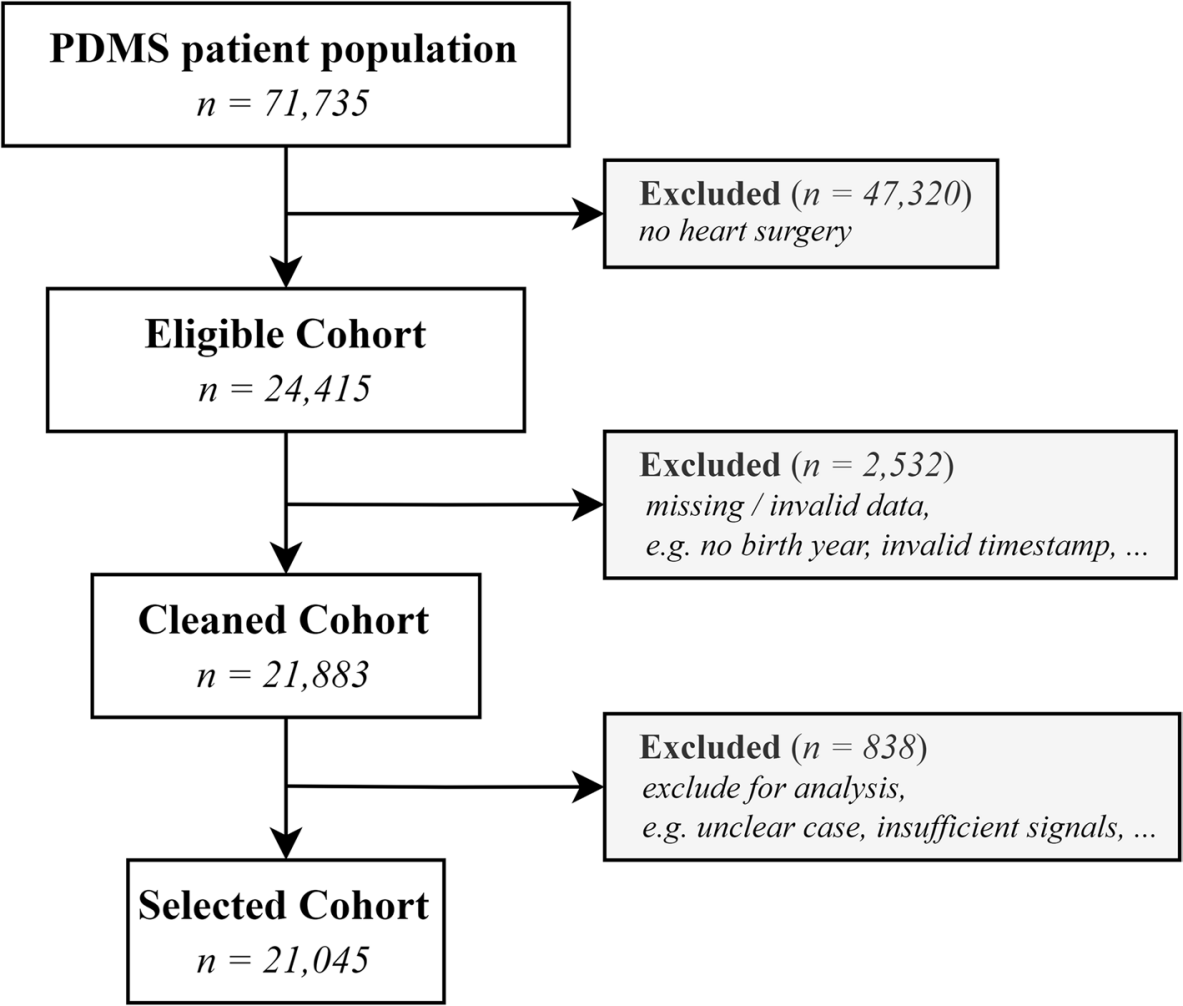
Summary of cohort generation. Patients were excluded with (1) surgery type other than heart related (2) with missing or invalid data, e.g. no birth year, inconsistent timestamp, unrealistic age, (3) improper data for use, e.g. unclear case, insufficient AKI signals, dialysis before ICU

**Fig. 2:**
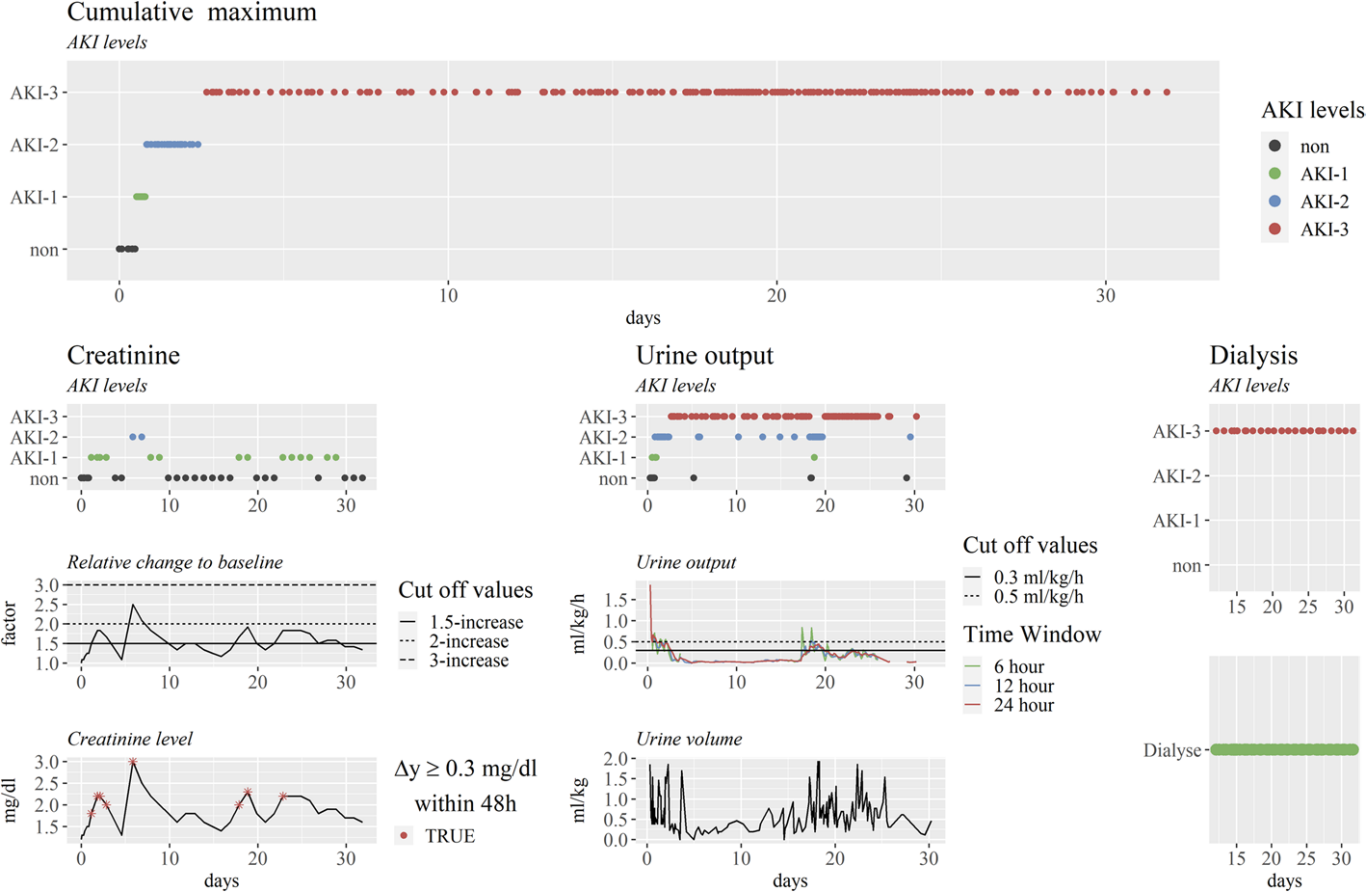
Visualization of the automated AKI detection process on an exemplary patient during ICU stay. In the first row the graphic depicts the cumulative maximum of the AKI detection by any information derived from the creatinine, urine and dialysis signal. Instead, the first row of the columns below shows the result of the AKI detection based on a single method. The remaining plots in each column from left to right show: the relative change in creatinine value from the selected baseline along with critical limits, the raw creatinine value along with red markers indicating critical increases from the observed minimum in the last 48 hours, the calculated urine output over different time windows in hours along with critical limits, the urine volume normalized by the body weight and a binary indicator of the need for dialysis. In all partial images, the x-axis describes the days since ICU admission

Primary data source for this work was the archive database of our internal digital patient documentation management system (PDMS). Data collected included general patient information (e.g. age, sex, weight), clinical (comorbidities, medication) and laboratory data. Unlike most related work, we used full KDIGO criteria in defining of AKI including urine output. Thus, we were not only dealing with data collected via automated transmission protocols using so-called Digiboxes or HL7 interfaces, but also with manually entered values. Nonetheless, to ensure high information quality, we preprocessed the extracted data in a three-step manner as depicted in Fig. 1, resulting in the described study population. Roughly speaking, patient exclusion criteria were (1) no documented heart surgery, (2) missing or invalid data and (3) improper data for use. Please find a detailed list of applied constraints in the supplemental appendix. Furthermore, as the risk factors and comorbidities were predominantly available as free-text signals, we implemented a mapping protocol concordant with International Classification of Diseases 10th Revision (ICD-10) Standards [12] for a set of AKI related diagnoses (see Supplemental Appendix). Finally, AKI stages were calculated at the time of every extracted urine, serum creatinine and dialysis related based on the corresponding rule. In doing so, urine outputs were normalized with (interpolated) body weights and derived for the required time windows. Regarding serum creatinine, we set the baseline to the first level obtained from our PDMS. Reasons were lacking of documentation of pre-surgery levels and the fact that creatinine levels shortly after surgery are unlikely to be greatly different from baseline level because of the time-dependent nature of accumulation of by-products in the absence of adequate kidney filtration. To better monitor the detection process, we further developed a Graphical User Interface (GUI) and combined all detections in a commutative manner (see Fig. 2). As we could not re-construct the exact time of ICU admission and discharge due to lack of documentation, each patient’s observation period commences with the first available timestamp of ICU signals across all available dynamic indicators (e.g. blood pressure, heart rate) and continues to the last signal within a period of 14 days. This approximates the total duration of ICU stay with an adequate degree of precision. Throughout this process, we ensure compliance with data protection regulations by de-identifying extracted information and referring to patients by numeric IDs. Technically, we used R v4.1.3 [13] for building the APIs and subsequent data analysis. Instead, database queries relied on joined statements built with Structured Query Language (SQL).

## Results

### Cohort description

The cohort consists of patients of the Robert Bosch Hospital (Stuttgart, Germany) admitted to the ICU after cardiac surgery between 2012-04-19 and 2022-07-14. After screening *N* = 24,415 potential study participants, we obtained a cohort comprising *N* = 21,045 distinct patients (see Fig. 1). Health records were analyzed for the first days following admission to the ICU (maximum 14 days), commencing with the datetime of the first available ICU signal. These records comprised data from both the ICU and intermediate care (IMC) as they both use the same PDMS and consequently the same database. As a result, the median (interquartile range (IQR)) time on ICU (including IMC) was 7.8 (5.8, 11.8) days. The patients were predominantly male (71.5%) and we found a median (IQR) age of 71 (62, 78) years at the time of ICU admission. Regarding patient’s comorbidities and risk factors related to AKI, 72.1% of patients had at least one of these documented, with chronic ischaemic heart disease (*N* = 10,678, 50.7%) and essential (primary) hypertension (*N* = 8693, 41.3%) being the most frequent. In turn, chronic kidney disease was evident in only 7.7% of the patients. Comparatively few patients received nephrotoxic medication, with Ibuprofen and Vancomycin being the most common, accounting for 2.6 and 2% of patients in the cohort, respectively. Please be referred to Table 2 and the supplemental appendix for details on the comorbidities and risk factors as well as on our assessment of nephrotoxic drugs.

**Table 2:** Comorbidities and risk factors dissected by subgroups after AKI detection

		Total cohort		Disease group			AKI stages		
Comorbidities/risk factors	ICD10	N	%	AKI (%) *N* = 11664	No AKI (%) *N* = 3516	***p***-value^a^	AKI 1 (%) *N* = 2848	AKI 2 (%) *N* = 5689	AKI 3 (%) *N* = 3127
Chronic ischaemic heart disease	I25	10,678	50.7	71.6	66.1	< 0.001	71.6	73.1	68.9
Essential (primary) hypertension	I10	8693	41.3	57.5	56.6	0.361	55.2	59.0	56.7
Disorders of lipoprotein metabolism and other lipidaemias	E78	4425	21.0	28.6	31.0	0.007	29.4	29.1	27.0
Unspecified diabetes mellitus	E14	3621	17.2	24.9	20.3	< 0.001	21.9	25.1	27.4
Nonrheumatic aortic valve disorders	I35	3458	16.4	23.4	20.8	0.002	23.1	23.5	23.3
Nonrheumatic mitral valve disorders	I34	2661	12.6	17.2	18.8	0.027	18.2	16.3	17.8
Atrial fibrillation and flutter	I48	2625	12.5	19.1	11.4	< 0.001	15.9	19.4	21.4
Chronic kidney disease	N18	1622	7.7	12.3	5.5	< 0.001	10.2	10.0	18.3
Obesity	E66	1533	7.3	11.0	7.1	< 0.001	7.2	12.1	12.4
Atherosclerosis	I70	1204	5.7	8.2	7.1	0.033	8.0	7.9	9.0
Other chronic obstructive pulmonary disease	J44	878	4.2	6.1	4.8	0.006	5.9	6.2	6.0
Sleep disorders	G47	548	2.6	3.9	2.6	< 0.001	2.9	4.2	4.2
Acute and subacute endocarditis	I33	508	2.4	3.7	2.2	< 0.001	2.7	3.1	5.7
Aortic aneurysm and dissection	I71	316	1.5	2.4	0.9	< 0.001	1.9	2.1	3.5

Note that except for total cohort, only patients with at least one considered comorbidity or risk factor are considered. Percentages are computed with N of each subgroup

^a^Pearson’s Chi-squared test

### Automated AKI detection

By our detailed analysis we were able to investigate the time and severity of each AKI event in great detail. Because different AKI criteria can be met several times within the period of ICU stay, after further analysis we distinguish between the first AKI (*first_AKI*) as the first detection of AKI within the observed ICU stay and further specify the time (*first_AKI_time*) and stage (*first_AKI_stage*) if necessary. In an analogous way, we denote the maximum AKI (*max_AKI*) as the time (*max_AKI_time*) and stage (*max_AKI_stage*) of the highest AKI level detected within a patient’s ICU stay. Overall, our implemented automatic AKI detection revealed *N* = 13,755 (65.4%) patients showing signs of at least AKI stage 1 or worse within the considered length of ICU stay and according to the international definition of KDIGO.

### Temporal course and development of the degrees of AKI severity

**Fig. 3:**
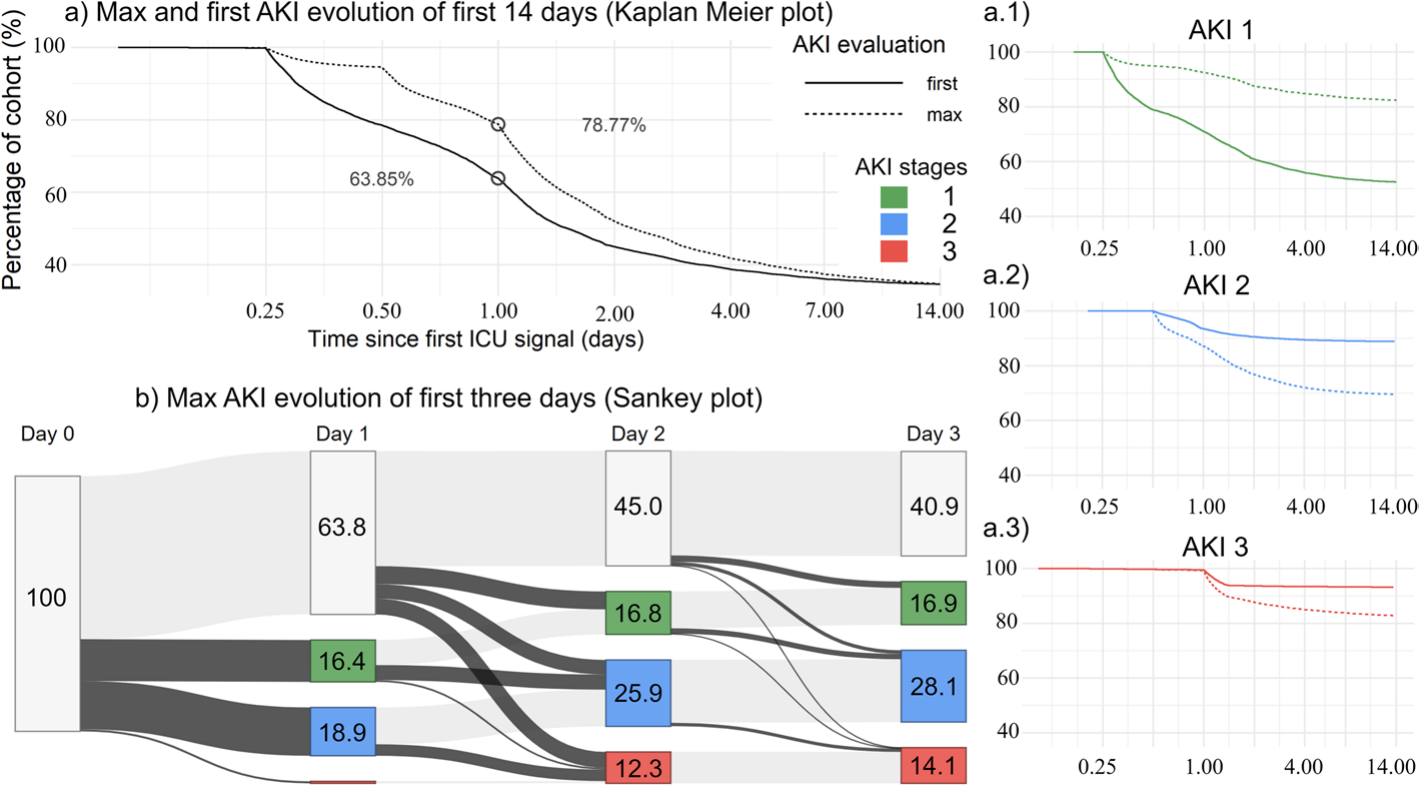
Evolution of AKI deterioration. **a** Kaplan Meier representation of first and max detected AKI over time both for any stage (**a**) and with respect to each individual stage (a.1–3). Here, max AKI does not refer to a specific day, but to the entire ICU time window of 14 days. For clarity, the x and y labels of the subfigures have been removed but can be taken from the main plot. **b** Sankey plot of max AKI evolution within first three days. Each bin represents the proportion of patients developing no (gray), 1 (green), 2 (blue) or 3 (red) as max AKI stage up to a specific day on ICU after admission. For clarity, bin values less than 1% have been removed

Regarding the patients’ first detection, the distribution of AKI stages was as follows: For *N* = 9984 patients (47.4%), AKI progression commenced with stage 1. In much lower proportions, AKI occurred initially already at stage 2 (11.1%) or stage 3 (6.8%). In terms of the patients’ *max_AKI_stage*, *N* = 6423 (30.5%) developed stage 2 as the final severity. Stage 1 and 3 were detected considerably less as *max_AKI_stage*, namely in merely 17.6 and 17.2% cases, respectively. Similarly, after dividing max AKI stages per days (see b) Fig. 3): Stage 2 was the most frequently observed AKI stage across the first 3 days (18.9, 25.9, 28.1%), followed by stage 1 and finally 3. Despite the relatively coarse temporal resolution of 24 hours, disease dynamics were clearly observable, i.e. on each day, a noticeable proportion of patients either initially developed AKI or exceeded their previous maximum stage. The latter explicitly suggests that disease progression in most AKI patients corresponded to a gradual deterioration with at least one intermediate stage. Particularly, only 14.4% of patients developed AKI 2 or 3 without a previously identifiable lower stage. As a result, the median number of days was 0.3 (stage 1 to stage 2, *N* = 4826), 1.3 (stage 1 to stage 3, *N* = 1451) and 0.5 (stage 2 to stage 3, *N* = 747).

Furthermore, Fig. 3 a) underlines that the magnitude of the temporal divergence between the first and maximum AKI stage is dependent on the window of examination. For 36.2% of the cohort, the first detection occurred within the first day, i.e. *first_AKI_time* was less than or equal to 1 day. Conversely, only 21.2% of patients already developed their max AKI stage within this timeframe. This discrepancy could potentially enable early therapeutic interventions, e. g. already evaluated KDIGO recommendation-based bundles [14], for 15% of patients before AKI progression continues beyond initial stages. Including the second day, however, the difference between patients who had already developed the first and maximum stages was reduced to 7% and continued to shrink in the subsequent days. When breaking down disease evolution into stages (see a.1–3) Fig. 3), we found that the proportion of patients developing AKI initially with a stage 2 or 3 after the second day is quite low. However, this is not true for the *max_AKI_stage*, which takes considerably more days to develop. Contrastingly, 17.4% of patients developing AKI 1 as initial stage show a *first_AKI_time* greater than 2 days. This further underlines the importance of automatic detection over several days.

### Comparison of AKI criteria

As we follow the international definition of acute kidney injury according to the KDIGO guidelines, our automated AKI detection relies on three different indicators: drop in urine output, rise in serum creatinine level and initiation of KRT (see Table 1). We assessed the relevance of these indicators using three metrics: total contribution and specifically the contribution to the detection of *max_AKI_stage* both generally and time-dependent. Overall, we found that the urine output indicator turned out to be the most relevant. Quantitatively, the criterion contributes in *N* = 12,004 cases, which is quite remarkable as it corresponds to 87.3% of the individual patients developing at least stage 1. In fact, in *N* = 11,665 patients, the urine criterion was the first criterion met when an AKI stage was first detected, followed by a triggered serum creatinine rule at a later timestep in 3950 cases. Moreover, in 55.9% of all patients, the *max_AKI_stage* was at some point detected by a drop in urine output (here, not necessarily exclusively). In 97.6% of these cases, the time of detection corresponds to *max_AKI_time*, which is to say whenever the urine criterion was met, it was most likely the criterion that was met first. To further substantiate these findings, Table 3 compares the AKI criteria divided by stages and in an exclusive manner. Strikingly, in 95.5% of cases where *max_AKI_stage* was AKI 2, *max_AKI_time* is analogous to the time the urine criteria needed to be met. The remaining 4.5% are solely attributable to a rise in creatinine level as the KRT rule directly and exclusively implies AKI 3.

**Table 3:** Comparison of AKI detection across each KDIGO criteria

	Disease group	AKI stages		
Characteristic	AKI (n, %) *N* = 13755	AKI 1 (n, %) *N* = 3707	AKI 2 (n, %) *N* = 6423	AKI 3 (n, %) *N* = 3625
Detection of max_AKI_stage				
Drop in urine output	**10,284 (75%)**	2023 (55%)	5862 (91%)	2399 (66%)
Rise in creatinine	**1845 (13%)**	1272 (34%)	273 (4.3%)	300 (8.3%)
Drop in urine output and rise in creatinine	**835 (6.1%)**	412 (11%)	288 (4.5%)	135 (3.7%)
Drop in urine output and initiation of dialysis and rise in creatinine	**334 (2.4%)**	0 (0%)	0 (0%)	334 (9.2%)
Drop in urine output and initiation of dialysis	**313 (2.3%)**	0 (0%)	0 (0%)	313 (8.6%)
Initiation of dialysis	**72 (0.5%)**	0 (0%)	0 (0%)	72 (2.0%)
Initiation of dialysis and rise in creatinine	**72 (0.5%)**	0 (0%)	0 (0%)	72 (2.0%)
First detection of max_AKI_stage				
Drop in urine output	**11,484 (83%)**	2388 (64%)	6132 (95%)	2964 (82%)
Rise in creatinine	**2032 (15%)**	1319 (36%)	291 (4.5%)	422 (12%)
Initiation of dialysis	**239 (1.7%)**	0 (0%)	0 (0%)	239 (6.6%)

Distinction is made between the general detection of a patient’s max AKI stage and the explicit time of detection. Percentages are computed with N of each subgroup

### Characterization of subgroups

In order to compare patients’ characteristics after applying automatic AKI detection, we divided our cohort into different (sub-) groups: AKI and no AKI group as well as a group for each possible *max_AKI_stage*. The significance of the discrepancy between the AKI and no AKI group was validated with either appropriate categorical or numerical statistical tests and reported by *p*-values with significance levels of 5% (see Table 4).

**Table 4:** Characterization of subgroups after AKI detection

	Total cohort	Disease group			AKI stages		
Characteristic	*N* = 21045 Median (IQR) / n (%)	AKI *N* = 13755 Median (IQR) / n (%)	No AKI *N* = 7290 Median (IQR) / n (%)	***p***-value^a^	AKI 1 *N* = 3707 Median (IQR) / n (%)	AKI 2 *N* = 6423 Median (IQR) / n (%)	AKI 3 *N* = 3625 Median (IQR) / n (%)
Age at admission	71 (62, 78)	72 (63, 78)	70 (61, 78)	< 0.001	72 (63, 78)	72 (63, 78)	72 (64, 78)
Male	15,048 (72%)	10,010 (73%)	5038 (69%)	< 0.001	2724 (73%)	4667 (73%)	2619 (72%)
Length ICU stay (day)	7.8 (5.8, 11.8)	8.8 (6.1, 12.5)	6.6 (4.9, 9.4)	< 0.001	7.6 (5.9, 11.1)	8.8 (6.2, 12.1)	10.1 (6.9, 13.5)
Number of drugs administered	18 (13, 24)	21 (16, 27)	13 (3, 18)	< 0.001	18 (14, 22)	21 (17, 27)	24 (18, 34)
Number of patients receiving nephrotoxic drugs	1374 (6.5%)	1097 (8.0%)	277 (3.8%)	< 0.001	200 (5.4%)	489 (7.6%)	408 (11%)
Number of different AKI comorbidities/ risk factors	2 (0, 3)	2 (1, 4)	0 (0, 2)	< 0.001	2 (1, 3)	3 (1, 4)	3 (1, 4)

Percentages are computed with N of each subgroup. Distinction is made between general patient info (age, sex), length of ICU stay, medication and comorbidities.

^a^Wilcoxon rank sum test; Pearson’s Chi-squared test

#### Patient info and ICU stay

With a median age at admission of 72 years (IQR 63, 78), patients developing AKI at any stage were significantly older than patients without detected AKI (*p* <  0.001). Similarly, there was a significant male predominance developing AKI. In contrast, we cannot report any clear age and gender differences with respect to a patient’s *max_aki_stage*. Not surprisingly, AKI patients showed a significantly longer ICU stay – median 2.1 days longer compared to the no AKI group (p <  0.001). Similarly, there is a significant increase in length of ICU stay (median days) when maximum stages are considered, starting with the lowest stage (7.6) up to AKI 3 (10.1).

#### Medication

Patients developing AKI seemed to receive a significantly higher median number (AKI, no AKI) of medication during ICU stay (21, 13). The difference in the number of patients receiving nephrotoxic drugs was significantly greater in the AKI group as well, at *N* = 1097 compared to *N* = 277 for patients without any AKI. Of far greater importance, however, is considering the administration of nephrotoxic medications with relation to the timing of AKI detections. We have found that the administration of nephrotoxic drugs after the first automatic detection of an AKI stage decreases as the condition progresses. That is, *N* = 574 patients received at least one nephrotoxic drug after the first detection of an AKI 1. By comparison, after the first detection of stage 2 and 3, we found *N* = 476 and *N* = 221 patients, respectively.

#### Comorbidities and risk factors

When comparing the mean number (IQR) of different comorbidities and risk factors considered, we found a significantly higher incidence in the AKI group: 2 (1, 4) compared to the group with no AKI with 0 (0, 2). The differences when incorporating the individual maximum levels were minor. Table 2 breaks down the frequency of each comorbidity and risk factor by the subgroups in 72.1% of patients having at least one documented comorbidity. Our intention to comprehensively outline comorbidities and risk factors related to AKI is supported by the fact that the incidence was significantly higher for patients developing AKI in almost all cases. Particularly, the greatest differences were seen in diseases of type aortic aneurysm and dissection and chronic kidney disease, which were more common in patients with AKI by a factor of 2.8 and 2.2, respectively.

### Underrepresentation in patient records

In contrast to the high incidence of detected AKI, we found evidence of explicitly documented AKI only for *N* = 375 (1.8%) patients within the corresponding PDMS records. This shows that the documented AKI diagnoses remarkably underrepresent the actual occurrence of AKI in the cohort by a factor of 36.7. However, it is noteworthy that in 38 incidents we could not reconcile the documented AKI with the results of automated detection. Reasons for this were: (1) incorrect parsed diagnoses caused by unconventional abbreviation such as aortic valve regurgitation (in german ‘Aortenklappeninsuffizienz’) as AKI; (2) documentation of previous diagnoses without time reference; (3) clinically determined AKI by indicators not related to KDIGO definition such as pathological findings postmortem examination. Nevertheless, our algorithm detected 89.9% of documented AKI cases.

## Discussion

To date, a comprehensive multi-faceted analysis relating to automated AKI detection in a large and defined ICU study population is scarce in the medical literature. Here we report on a successful implementation of algorithm-based AKI detection, which we applied to a large collective of postoperative cardiac surgical intensive care patients. To the best of our knowledge, no data exist to date on postoperative AKI after cardiac surgery in this large number of cases with inclusion of urinary output. In this study, we could demonstrate the following:

The overall incidence of AKI after cardiac surgery is as high as 65%. Most reported AKI rates range between 20 and 40% depending on AKI definition and criteria [15–19]; studies employing the complete KDIGO criteria [20, 21] showed similar AKI rates using the KDIGO definition and including urine output as criterion. In fact, we demonstrated that urine output is of great importance as one of the earliest clinical indicators for the development of AKI. Moreover, urine output was also the most frequent trigger in our automated AKI detection model. We found urine output to be a far more relevant indicator for the onset and subsequent evolution of AKI compared to other parameters such as serum creatinine levels and onset of KRT. This is in accordance with current literature [22] and reflects the known limitation of the static kidney function marker creatinine. Of note, onset of KRT defines high-grade kidney failure but provides little applicability in preventing AKI. Serum creatinine levels are relevant but depend on the frequency and consistency of bloodwork. While it is performed relatively frequently on a monitoring ward, urine output has a much better temporal resolution because it is registered on an hourly basis and can provide a more dynamic overview. As every cardiac surgical patient receives a urinary catheter prior to surgery monitoring output is relatively straightforward and should provide no hindrance in identifying AKI. The only source of potential error is the human factor, as these values are manually registered. Finally, the comparison of AKI criteria further showed that in the majority of cases the urine criterion was met first for AKI detection. In slightly more than one-third of these cases, a subsequent increase in creatinine levels was observed to such an extent that the creatinine criteria was met as well. This reinforces the fact that urine output - although quite unspecific - is an early marker of imminent AKI.

The relevance of AKI in intensive care medicine, especially following cardiac surgery, cannot be over-emphasized as it has been proven by a variety of publications to greatly elevate mortality, major adverse cardiac and cerebrovascular events (MACCE) rates and length of ICU and hospital stay [15–20, 22, 23]. Therefore, early detection is the cornerstone and first prerequisite of any preventive or therapeutic strategies. Timely intervention has the potential to reduce the incidence and severity of postoperative AKI as recommended by KDIGO [14, 24].

In the majority of patients, there is a considerable time window between developing their first and maximum AKI stages, especially when including urine output data, so most cases showed a gradual disease progression after onset. With this time interval, early diagnosis allows for the application of nephroprotective care bundles with the potential to prevent or attenuate a progressive decline of kidney function [19, 24]. To enable early intervention increasing AKI awareness is essential. Based on the present data there is a huge discrepancy between documented cases of acute kidney injury and automatically detected cases (1.8% vs. 65.4%). As outlined above, this automated detection provided a very high accuracy rate in the patient population. Such underrepresentation, most likely due to inadequate detection, has recently been reported both in overall patient populations and in a post-cardiac surgery setting [9, 25]. Detecting AKI-defining, slight decreases of urine output can be challenging as in clinical routine urine output is mostly not documented in relation to body weight and changes can be overseen quickly. Automated detection can alert physicians, reduce workload and provide assistance in an otherwise intricate multifaceted diagnostic and therapeutic process. Furthermore, the definition and automated detection of AKI can serve as a starting point for further developments including machine-learning based prediction algorithms that can calculate the risk of developing an AKI far before any clinical manifestations are present [3].

Our data underline the detrimental effects of AKI: ICU stay is prolonged significantly in AKI patients with a consequent stage-dependent increase up to median 10 days in cases requiring KRT. Frequently associated secondary complications such as volume overload, low cardiac output syndrome, edemas, respiratory failure and infection result in an elevated risk of morbidity and mortality as well as greatly increased treatment costs [23, 26]. However, the consequences are not limited to the individual patient, as shortening ICU stays and lowering complication rates can ultimately reduce workload and costs and increase efficiency of the intensive care unit as a whole. The global need for a responsible distribution of sometimes limited material and human resources became all too obvious in the previous years because of the COVID pandemic crisis.

We also found our AKI patients received more medication (including nephrotoxic drugs). A causative association cannot be proven as the overall quantity of prescribed medication might only be a surrogate indicator for the severity of a patient’s condition which can itself carry a higher risk of developing AKI. Moreover, the impact of nephrotoxic drugs on AKI progression has to be taken into consideration from a cost/benefit perspective related to patient care, as some nephrotoxic drugs are essential for treating or preventing diseases that pose a more serious threat than kidney failure, e.g. antibiotics in endocarditis. Nonetheless, early detection of AKI can facilitate a better-tailored multidisciplinary medication and treatment concept, with the timely identification and modification/reduction of non-essential or replaceable potentially nephrotoxic drugs.

### Limitations

Due to the used methods, some limitations have to be discussed. Errors in the assignment of inclusion criteria may have occurred due to automatic case detection. However, we could not detect an increased error rate in random manual sampling. Furthermore, our approach facilitated the study of a very large collective over a long period of time, which compensates for possible isolated errors. Regarding the coded diagnoses, undocumented AKI does not automatically exclude clinical diagnosis. However, such large discrepancies can only be attributed to non-recognition. In addition, another (manual) evaluation by the study group also showed significant under-diagnosing of postoperative kidney injury [9]. In terms of data quality for urine output documentation, we put a lot of effort into evaluating the data quality. In average (median) about 10.6 urine output observations were documented per patient and day. Furthermore, 95% off all patients had less than 4.5 hours (on average) between two urine observations and hence less than the 6 h time window from the definition. However of course, it is clear that in individual cases incorrect or missing documentation of the urine output influences AKI detection. Nevertheless, we could not find any indications of a systematically missing documentation. Furthermore, this study is of retrospective nature, therefore causal relationships and the actual clinical impact of AKI detection cannot be investigated.

### Future perspectives

Based on the developed AKI detection algorithm, a prediction model is under development. This model will be prospectively evaluated and ultimately introduced into clinical practice.

## Conclusions

We demonstrated that algorithm-based detection of AKI is feasible, and demonstrated exceptionally reliable detection of AKI after cardiac surgery. This resulted in a strikingly high rate of AKI recognition in our patient cohort compared to conventional routine documentation. Automated AKI detection has the potential to facilitate early therapeutic intervention to prevent or attenuate disease progression and improve patient outcomes.

## Supplementary Information

**Additional file 1: Supplemental Appendix. Table S1.** Diagnosis parsing from free text to ICD-10 codes: Accuracy is assessed by manually detecting incorrect assignments for each of the 15 diagnoses for 400 randomly selected free-text diagnoses. Accuracy considers true positives and negatives. **Table S2.** Distribution over nephrotoxic drug within the cohort. Absolute number of patients and percentages are given. Multiple administration of the same drug for a patient is not considered. **Table S3.** Summary of applied cleaning and selection steps during data processing. While the former ensures data consistency, the latter selects relevant data which is then forwarded to AKI detection.

## Data Availability

The datasets used and analysed during the current study are available from the corresponding author on reasonable request after internal board review.

## Abbreviations

AI: Artificial intelligence
AKI: Acute kidney injury
API: Application programming interface
ICD-10: International Classification of Diseases 10th Revision
ICU: Intensive care unit
IMC: Intermediate care
IQR: Interquartile range
KDIGO: Kidney Disease: Improving Global Outcomes
KRT: Kidney replacing therapy
MACCE: Major adverse cardiac and cerebrovascular events
PDMS: Patient documentation management system
SQL: Structured Query Language
