# Supplementary material for: Algorithm-based detection of acute kidney injury according to full KDIGO criteria including urine output following cardiac surgery: a descriptive analysis
Source: BioData Min. 2023 Mar 16;16:12. doi: 10.1186/s13040-023-00323-3 (PMC10022284; doi:10.1186/s13040-023-00323-3)
Supplement: Supplementary file 1 — Additional file 1: Supplemental Appendix. Table S1. Diagnosis parsing from free text to ICD-10 codes: Accuracy is assessed by manually detecting incorrect assignments for each of the 15 diagnoses for 400 randomly selected free-text diagnoses. Accuracy considers true positives and negatives. Table S2. Distribution over nephrotoxic drug within the cohort. Absolute number of patients and percentages are given. Multiple administration of the same drug for a patient is not considered. Table S3. Summary of applied cleaning and selection steps during data processing. While the former ensures data consistency, the latter selects relevant data which is then forwarded to AKI detection. [file 13040_2023_323_MOESM1_ESM.docx]

# Supplemental Appendix

## Parsing of Diagnoses

Generally, unstructured data is not unusual in clinical environments, which makes mapping and filtering steps usually essential to use said data to its fullest potential. This also includes the considered diagnoses, as they are predominantly available as free-text signals within our PDMS, providing a high degree of freedom for physicians, however, complicating automated data extraction because of non-standard abbreviations, repeated single spelling mistakes, word compounds and circumlocution. While natural language processing and algorithms for intelligent keyword retrieval have been an emerging field in recent years and have produced impressive accomplishments, we found a rather manual approach better suited to address the aforementioned peculiarities of the underlying text corpus. In doing so, we have implemented a mapping protocol from free-text to 15 ICD-10 encoded diagnoses. In particular, we define abbreviations, synonyms, misspells and build regular expressions to assign free-text inputs to one or multiple of the aforementioned diagnoses. Through several rounds of discussion and re-evaluation, we iteratively improved the accuracy of the mapping and resolved disagreements, achieving a general accuracy of 0.994% on 400 randomly chosen free-text diagnoses, which yields 6,000 potential true positives or negatives (6,000, as for each of the 400 free-text diagnoses a subset of the 15 considered ICD-10 diagnoses may be assigned). In absolute terms, our mapping assigns the wrong diagnoses in merely 37 out of the said 6,000 cases. For assessing accuracies per diagnosis, please be referred to Table S1.


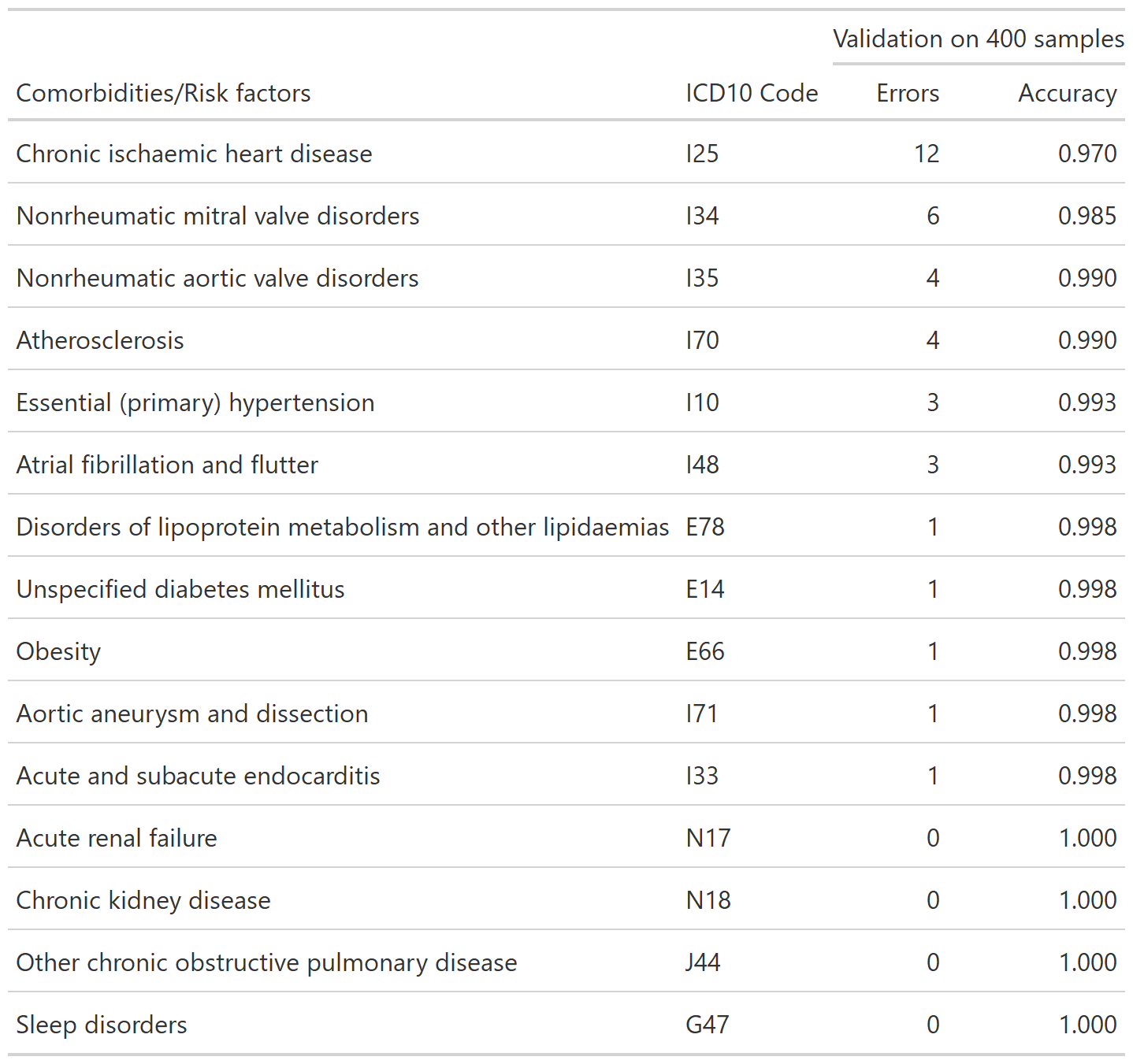


Table S1: Diagnosis parsing from free text to ICD-10 codes: Accuracy is assessed by manually detecting incorrect assignments for each of the 15 diagnoses for 400 randomly selected free-text diagnoses. Accuracy considers true positives and negatives.

## Definition of Nephrotoxic Medication

We consider the following drugs as nephrotoxic: Ibuprofen, Vancomycin, Gentamicin, Diclofenac, Tobramycin (Tobrazid), Foscarnet, Ampho B, Amphotericin B (Liposomal), Celecoxib, Emtricitabin / Tenofovirdisoproxil, Teicoplanin, Cisplatin and Carboplatin. We are well aware of other papers that have defined this list more extensively, including more interventions that may have a negative impact on kidney health. To the best of our knowledge, however, we found that the causal relationship in some cases is not entirely clear. For example, in the case of blood transfusions, the fact that an individual requires such an intervention is the actual causal factor for possible subsequent kidney disease, not the documented intervention itself. Therefore, we only list medications for which we suspect causal relationships with a relatively high degree of certainty. See figure S2 for the distribution of the mentioned drugs within the cohort.


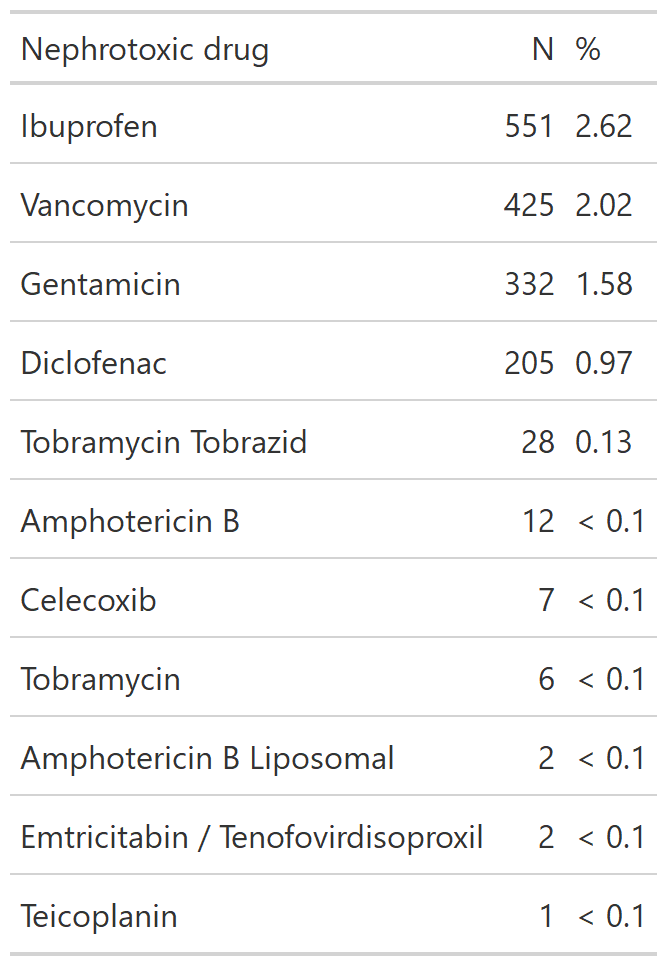


Table S2: Distribution over nephrotoxic drug within the cohort. Absolute number of patients and percentages are given. Multiple administration of the same drug for a patient is not considered.

## Data Exclusions


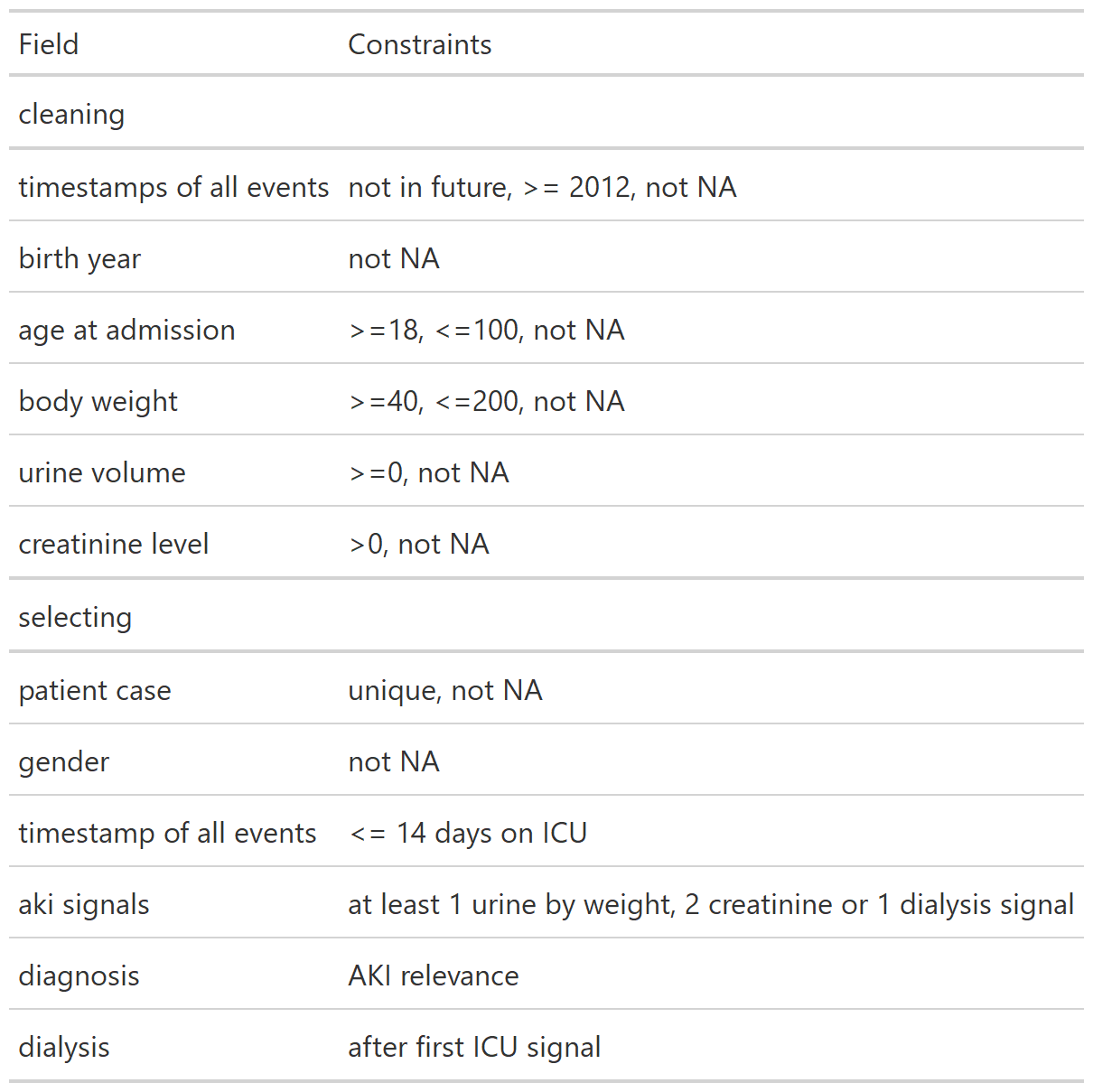


Table S3: Summary of applied cleaning and selection steps during data processing. While the former ensures data consistency, the latter selects relevant data which is then forwarded to AKI detection.
